# Expert consensus statement on therapeutic drug monitoring and individualization of linezolid

**DOI:** 10.3389/fpubh.2022.967311

**Published:** 2022-08-10

**Authors:** Bin Lin, Yangmin Hu, Ping Xu, Tao Xu, Chunyan Chen, Le He, Mi Zhou, Zhangzhang Chen, Chunhong Zhang, Xuben Yu, Luo Fang, Junfeng Zhu, Yanlan Ji, Qun Lin, Hengbin Cao, Youqin Dai, Xiaoyan Lu, Changcheng Shi, Li Li, Changjiang Wang, Xumei Li, Qiongyan Fang, Jing Miao, Zhengyi Zhu, Guangyong Lin, Haichao Zhan, Shiwen Lv, Yalan Zhu, Xinjun Cai, Yin Ying, Meng Chen, Qiong Xu, Yiwen Zhang, Yubin Xu, Federico Pea, Saiping Jiang, Haibin Dai

**Affiliations:** ^1^Department of Pharmacy, Changxing People's Hospital, Changxing Branch, Second Affiliated Hospital of Zhejiang University School of Medicine, Huzhou, China; ^2^Key Laboratory of Intelligent Pharmacy and Individualized Therapy of Huzhou, Huzhou, China; ^3^Department of Pharmacy, Second Affiliated Hospital of Zhejiang University School of Medicine, Hangzhou, China; ^4^Department of Pharmacy, Ningbo First Hospital, Ningbo, China; ^5^Department of Pharmacy, Ruijin Hospital, Shanghai Jiao Tong University School of Medicine, Shanghai, China; ^6^Department of Pharmacy, Children's Hospital of Soochow University, Suzhou, China; ^7^Department of Pharmacy, Zhongshan Hospital, Fudan University, Shanghai, China; ^8^Department of Pharmacy, The First Affiliated Hospital of Wenzhou Medical University, Wenzhou, China; ^9^Department of Pharmacy, The Cancer Hospital of the University of Chinese Academy of Sciences (Zhejiang Cancer Hospital), Institute of Basic Medicine and Cancer (IBMC), Chinese Academy of Sciences, Hangzhou, China; ^10^Department of Pharmacy, Deqing People's Hospital, Huzhou, China; ^11^Department of Pharmacy, Tiantai People's Hospital, Taizhou, China; ^12^Department of Clinical Pharmacy, Huzhou Central Hospital, Huzhou, China; ^13^Department of Pharmacy, Hwa Mei Hospital, University of Chinese Academy of Sciences, Ningbo, China; ^14^Department of Clinical Pharmacy, Key Laboratory of Clinical Cancer Pharmacology and Toxicology Research of Zhejiang Province, Affiliated Hangzhou First People's Hospital, Zhejiang University School of Medicine, Hangzhou, China; ^15^Department of Pharmacy, Zhejiang Hospital, Hangzhou, China; ^16^Department of Pharmacy, The Second Affiliated Hospital of Jiaxing University, Jiaxing, China; ^17^Department of Pharmacy, Zhoushan Hospital, Zhoushan, China; ^18^Department of Pharmacy, The Children's Hospital, Zhejiang University School of Medicine, National Clinical Research Center for Child Health, Hangzhou, China; ^19^Department of Pharmacy, The Second Affiliated Hospital of Wenzhou Medical University, Wenzhou, China; ^20^Department of Clinical Pharmacy, Affiliated Jinhua Hospital, Zhejiang University School of Medicine, Jinhua, China; ^21^Department of Pharmacy, Affiliated Hangzhou Chest Hospital, Zhejiang University School of Medicine, Hangzhou, China; ^22^Department of Pharmacy, Tongde Hospital of Zhejiang Province, Hangzhou, China; ^23^Department of Pharmacy, The First Hospital of Jiaxing, Affiliated Hospital of Jiaxing University, Jiaxing, China; ^24^Department of Pharmacy, Putuo Hospital, Zhoushan, China; ^25^Clinical Pharmacy Center, Department of Pharmacy, Zhejiang Provincial People's Hospital, Affiliated People's Hospital, Hangzhou Medical College, Hangzhou, China; ^26^Department of Pharmacy, Taizhou Central Hospital (Taizhou University Hospital), Taizhou, China; ^27^Department of Medical and Surgical Sciences, Alma Mater Studiorum, University of Bologna, Bologna, Italy; ^28^SSD Clinical Pharmacology, Department for Integrated Infectious Risk Management, IRCCS Azienda Ospedaliero-Universitaria di Bologna, Bologna, Italy; ^29^Department of Clinical Pharmacy, First Affiliated Hospital of Zhejiang University School of Medicine, Hangzhou, China

**Keywords:** linezolid, therapeutic drug monitoring, individualization, expert consensus, pharmacotherapy

## Abstract

Linezolid is an oxazolidinone antibacterial drug, and its therapeutic drug monitoring and individualized treatment have been challenged since its approval. With the in-depth clinical research of linezolid, we have changed our attitude toward its therapeutic drug monitoring and our view of individualized treatment. On the basis of summarizing the existing clinical studies, and based on the practical experience of each expert in their respective professional fields, we have formed this expert consensus. Our team of specialists is a multidisciplinary team that includes pharmacotherapists, clinical pharmacology specialists, critical care medicine specialists, respiratory specialists, infectious disease specialists, emergency medicine specialists and more. We are committed to the safe and effective use of linezolid in patients in need, and the promotion of its therapeutic drug monitoring.

## Introduction

Linezolid is a synthetic antibacterial drug of the oxazolidinone class. It was approved by the U.S. Food and Drug Administration (FDA) in 2000 and entered the Chinese market in 2007 for clinical use (1, 2). Linezolid inhibits protein synthesis by binding at the P site of the ribosomal 50S subunit. In terms of pharmacokinetics (PK), it has good penetration, and its accumulation in tissue—including bone, lung, vegetation, hematoma, and cerebrospinal fluid (CSF)—allows for its use in treating surgical infection (3–7). However, differences in linezolid exposure between individuals are related to differences in efficacy and adverse reactions (8–12).

Currently there is no guideline or consensus for linezolid therapeutic drug monitoring (TDM) and individualized treatment. Therefore, the Division of Therapeutic Drug Monitoring of Zhejiang Pharmacological Society and the Infectious Diseases Pharmacist Group of the Society of Hospital Pharmacy of Zhejiang Pharmaceutical Association recruited experts in related fields to discuss issues related to linezolid TDM and individualized treatment. The expert group considered 18 clinical practice issues and conducted three rounds of consultation with external experts; the end result is this consensus statement.

## Methods

The expert consensus was drawn up by 36 pharmacotherapy experts from the Division of Therapeutic Drug Monitoring of Zhejiang Pharmacological Society and the Infectious Diseases Pharmacist Group of the Society of Hospital Pharmacy of Zhejiang Pharmaceutical Association. The group's agenda was predefined. The expert group first defined the clinical questions to be addressed and then designated experts to each question. The recommendations for the clinical practice questions are in a question-and-answer format and reasons are provided. Three rounds of expert meetings were conducted to provide trustworthy recommendations.

Eighteen external clinicians and experts in clinical pharmacotherapeutics were invited to vote on the issues. Scoring was based on theoretical basis, scientificity, innovation, feasibility, and expert weighting of the consensus items. According to the modified Delphi method (13), the voting results of experts are summarized, and the average score is calculated to propose the corresponding recommendation strength. The final score was on a scale from 1 to 10: 1–4 is not recommended, 5–7 is weakly recommended, and 8–10 is recommended.

## Expert panel recommendations

### Question 1: Does linezolid require TDM? What is the target range for linezolid TDM?

#### Expert panel recommendations

(1) We recommend TDM for linezolid. (Score: 8.90) (2) We recommend maintaining a linezolid trough concentration of 2–8 mg/L. (Score: 8.60)

#### Reason

A decade-long retrospective study by Pea et al. included 1,049 patients treated with linezolid 600 mg q12h and collected 2,484 trough concentration points. They set the trough concentration range to 2–7 mg/L. Only 50.8% of patients were in this range, and the incidence of overexposure (33%) was significantly higher than that of underexposure (16.2%). Overexposure to linezolid is significantly correlated with an estimated Creatinine clearance (CrCL) was estimated by means of the Cockcroft and Gault formula (CrCL_C−G_) ≤ 40 mL/min, and underexposure is significantly correlated with an estimated CrCL_C−G_ > 100 mL/min. This suggests that TDM could be used to optimize linezolid exposure in most patients (14). A monocentric, prospective, open-label, interventional study found that proactive TDM of linezolid may be beneficial for preventing or recovering from dose-dependent thrombocytopenia (15). A prospective observational study enrolled 84 patients who received the standard linezolid regimen (600 mg q12h) and collected 153 trough concentration points. Only 57.52% of patients had trough concentrations within the effective range (2–8 mg/L), 31.37% had underexposed trough levels, and 11.11% had overexposed trough levels (16). A retrospective, single-center, observational trial from 2008 to 2013 included 70 patients treated with linezolid 600 mg q12h. A linezolid trough concentration of 2–6.3 mg/L achieved the best therapeutic effect while minimizing adverse reactions (17). Another retrospective study included 108 patients treated with linezolid 600 mg q12h. Multivariate analyses showed that the clinical failure rate of the TDM group was significantly lower than that of the non-TDM group. In addition, 90.5% of patients with renal impairment in the TDM group needed a dose adjustment to achieve the target trough concentration (18). A prospective study by Cattaneo et al. (19) suggested a causal relationship between the blood concentration of linezolid and the risk for drug-related hematological toxicity. In addition, case reports of two critically ill patients with normal renal function suggested that overexposure to linezolid in plasma causes toxicity and that subtherapeutic exposure leads to treatment failure. TDM would allow early detection and correction of bias to achieve the target linezolid concentration (20).

In short, both prospective and retrospective trials and case reports suggest that TDM of linezolid is necessary. Although this is contrary to the description in the package insert of linezolid, it is a confirmed, evidence-based recommendation. We recommend that in patients treated with linezolid, in particular critically ill patients, TDM is required to achieve optimal PK.

### Question 2: What detection method is recommended for TDM of linezolid?

#### Expert panel recommendations

We recommended quantification of linezolid in human plasma or serum by high performance liquid chromatography-Ultraviolet detector (HPLC-UV) or liquid chromatography-tandem mass spectrometry (LC-MS/MS) methods. (Score: 8.80)

#### Reason

HPLC-based methods coupled with UV or MS are the major analytical techniques for determining linezolid in human plasma or serum, and MS is more suitable for clinical application because of its high selectivity and sensitivity (21). LC-MS/MS methods that enable simultaneous quantification of plasma concentrations of linezolid and other antibiotics are available (22–27) and may improve detection efficiency for TDM. Other methods for determining linezolid in human plasma are immunoassay (28), direct analysis in real time mass spectrometry (DART-MS) (29), and square wave voltammetry (30), but these lack comparative verification with HPLC for large-scale clinical sample detection.

In summary, based on the stability of the quantitative method and the availability of TDM equipment, we recommend HPLC-UV or LC-MS/MS methods for quantitative determination of linezolid in plasma.

### Question 3: How should laboratories conducting quantitative determination of linezolid perform quality control?

#### Expert panel recommendations

Quality control procedures should be implemented using drug *in vivo* analysis techniques, quality control standards, and clinical intervention programs. (Score: 9.15)

#### Reason

The quality control of linezolid by *in vivo* drug analysis techniques should include investigations of specificity, sensitivity, accuracy, reproducibility, and stability (22–27). Quality control standards should include indicators for quantitative determination methods within and between laboratories, qualifications and certification of testing personnel, Standard Operating Procedure (SOPs) for testing procedures, clinical pathways and independent external quality assessments (EQAs) (31). The laboratory should have a dedicated quality control manager. The laboratory should participate in the quality evaluation activities of the TDM professional organization or government-authorized quality management agency and have sufficient capacity. For quantitative detection of linezolid, relevant technical guidance documents, quality control plans, and clinical intervention plans should be formulated in advance. These quality control documents should be released after review by the appropriate authorities before quantitative testing of linezolid (31).

### Question 4: How should linezolid TDM results be reported?

#### Expert panel recommendations

We recommend that the TDM report provide test results, explanations thereof, and treatment suggestions. The report should be individualized for the patient in question. (Score: 9.15)

#### Reason

There is no best practice for TDM reports, but we base these recommendations on *Antimicrobial Therapeutic Drug Monitoring in Critically Ill Adult Patients: A Position Paper, The Expert Consensus on the Standards of Therapeutic Drug Monitoring (2019 Edition)*, and clinical practice experience. A complete linezolid TDM report should include basic information, test results, and treatment suggestions and explanations (31–33). Basic information includes information on the applicant, patient, and sample. Patient information should include the type of patient, identification, sex, age, weight, serum creatinine level, and estimated creatinine clearance rate (CLCr) on the day of sampling or the past 3 days. The minimum inhibitory concentration (MIC) value of pathogens is crucial, and should include confirmed or suspected pathogens and local drug resistance information for subsequent pharmacokinetic/pharmacodynamic (PK/PD) calculations. The PK of linezolid is affected by, for example, age, sex, weight, renal function, and rifampin contamination. Therefore, these factors should be included in the TDM report. Sample information should include the type of sample, collection time, and linezolid regimen (dose and time of administration of the last two doses). Only the qualified samples make the interpretation of significance. Any errors in the timing of specimen collection, collection of blood in arteries or veins or central venous catheter (CVCs), blood collection tubes, etc., can mislead the results.

Test results should include the concentration, quantitative unit, therapeutic range, and over/under limit mark. The interpretation of the test results should include the purpose of monitoring, an analysis of the results, and a conclusion. The conclusion should integrate the regimen, renal function, contaminant medication, and clinical response. Treatment suggestions should be based on the test results, purpose of monitoring, and patient information. If the linezolid dosage needs to be adjusted, we recommend pharmacometrics-based dose calculation. Pharmacometrics enables individualized administration of linezolid (8, 10). In addition, individualized monitoring and follow-up are needed based on characteristics of the patient and disease.

The TDM report should be issued within 24 h to provide time to adjust treatments. The report should contain the signatures of the test operator and the reviewer of the report.

### Question 5: For which patients should linezolid TDM be considered?

#### Expert panel recommendations

We recommend TDM for critically ill patients, children, patients with renal insufficiency/augment or liver cirrhosis, elderly, obese and patients taking co-medications known to interact with linezolid. (Score: 9.50).

#### Reason

Increased endothelial permeability, renal dysfunction, and hypoalbuminemia are common in critically ill patients. Endothelial dysfunction may increase the distribution volume of hydrophilic antimicrobials. Hypoalbuminemia may increase the free (or unbound) fraction of antimicrobials, potentially resulting in CL and increasing the drug distribution (34, 35). ARC (CrCL > 130 mL/min) is increasingly noted in critically ill subpopulations (incidence: 14–80%). It could enhance renal elimination of antimicrobials and is associated with suboptimal plasma concentrations (36–38). Many critically ill patients have renal impairment or acute kidney injury. All these factors will significantly influence antimicrobial PK in critically ill patients. Therefore, TDM should be routinely performed when linezolid is used to treat critically ill patients. Maintaining a linezolid C_min_ of 2–7 mg/L is recommended to optimize efficacy and minimize hematological toxicity (33). A retrospective, monocenter, observational study by Dong et al. included 70 critically ill patients treated with linezolid 600 mg q12h. Patients were divided into two groups according to whether they developed thrombocytopenia after treatment with linezolid. Logistic analyses showed that C_min_ was significantly related to linezolid-associated thrombocytopenia (17). Two other studies (39, 40) showed that linezolid overexposure in patients with renal insufficiency is related to thrombocytopenia and that linezolid blood concentration monitoring can avoid platelet toxicity caused by overexposure. A retrospective case-control 1:1 study by Luque et al. included 52 patients with and without cirrhosis who received linezolid 600 mg intravenously every 12 h. Patients with liver cirrhosis had higher median linezolid trough plasma concentrations than those without cirrhosis (20.6 mg/L vs. 2.7 mg/L, *P* < 0.001) and a significantly increased incidence of overexposure (76.9% vs. 26.9%, *P* < 0.001). Therefore, liver cirrhosis may influence linezolid PK. TDM of linezolid would be valuable in these patients (41).

A single-center retrospective study by Cojutti et al. included 112 overweight and obese patients on linezolid with a median body mass index (BMI) of 35.4 kg/m^2^ and a median weight of 105.8 kg. A total of 52.9% patients who received the standard dosage of 600 mg q12h did not achieve the target steady-state trough linezolid concentration (2–7 mg/L). Indeed, the trough linezolid concentration was <2 mg/L and >7 mg/L in 35.6% and 17.3% of patients, respectively (42). Therefore, the standard dose of linezolid may not be applicable to obese and overweight patients. TDM is needed in such patients to optimize treatment. Simon et al. (43) suggested that as body weight increases the linezolid concentration in plasma and subcutaneous tissue decreases. The current dosing regimen does not achieve a sufficient concentration to kill bacteria with MIC ≥ 2 mg/L, in particular as an empirical antibacterial for severely obese patients. Blackman et al. showed that for 140 kg non-cirrhotic patients, bacteria had MICs of 0.5, 1, 2, and 4 mg/L, and the standard linezolid dose of probability of target attainment (PTA) was 100, 98.8, 34.1, and 0%. For non-cirrhotic patients of body weight ≥ 140 kg and MIC ≥ 2 mg/L, the standard linezolid dose did not reach the target of ≥90%. The standard linezolid dose may not be suitable for all patients (44).

A retrospective study of 23 patients was conducted by Cojutti et al. Standard dosages were suboptimal in 50.0 and 44.4% of patients in group 1 and group 2, respectively. Among those who underwent multiple instances of TDM, the dosage was increased in 33.3% of cases in both groups and decreased in 6.6 and 9.5% of cases in group 1 and group 2, respectively. Monte Carlo simulations showed PTA ≥ 90% with the current dosing regimens in both groups for pathogens with MIC ≤ 1 mg/L. Therefore, the standard dose of linezolid may not be applicable to pediatric patients. Such patients require TDM to optimize treatment (9). Rao et al. suggested that an AUC:MIC ratio of 80–100 is an appropriate efficacy threshold for children, who tend to clear linezolid significantly faster than adults. However, the threshold for toxicity is less well defined in pediatric patients than in adults (45).

Linezolid is a potential substrate of P-glycoprotein, which interacts with numerous drugs. The putative mechanism underlying such interactions is modulation of P-glycoprotein activity. Clarithromycin, proton pump inhibitors (such as omeprazole and pantoprazole), amiodarone, amlodipine, and calcium channel blockers inhibit P-glycoprotein and therefore increase linezolid concentrations. Rifampin, phenobarbital, and levothyroxine are inducers of P-glycoprotein that increase clearance of linezolid and decrease linezolid plasma concentrations (9, 45–47). Therefore, TDM is required when linezolid is combined with the aforementioned drugs.

### Question 6: How should the linezolid dosage be adjusted for patients with renal insufficiency?

#### Expert panel recommendations

(1) For patients with renal insufficiency not on hemodialysis (HD), we suggest that linezolid can be reduced to a regimen of 300 mg q12h. (Score: 7.90) (2) Insufficient linezolid doses in patients with renal insufficiency on dialysis must be considered, and the dose should be increased based on TDM if necessary. (Score: 8.70) (3) Irrespective of whether patients with renal insufficiency are on dialysis, we recommend adjusting the dose of linezolid based on TDM. (Score: 8.15)

#### Reason

Pea et al. (14) suggested that overexposure to linezolid is significantly associated with renal insufficiency (Ccr ≤ 40 mL/min). A prospective observational study by Fang et al. (16) showed that renal insufficiency (Ccr ≤ 40 mL/min) was significantly associated with a linezolid trough concentration > 8 mg/L. A Spanish retrospective study showed that the risk of overexposure for estimated glomerular filtration rate (eGFR) <40 mL/min is 4.27-fold that of eGFR > 80 mL/min (48). Souza et al. (40) reported that the median linezolid concentration in patients with renal insufficiency was 1.46-fold that of patients with normal function. In summary, renal insufficiency is closely related to linezolid overexposure.

Crass et al. constructed a population pharmacokinetic (PPK) model based on 603 patients on linezolid. For patients with eGFR <60 mL/min, the risk of overexposure (C_min_ > 8 mg/L) to linezolid at the conventional dosage of 600 mg q12h was >50%. Linezolid 300 mg q12h can balance safety and effectiveness and seems to be feasible (39). The PPK/pharmacodynamic model of Sasaki et al. revealed that for Ccr ≤ 30 mL/min, 300 mg q12h linezolid is >90% likely to achieve the pharmacodynamic target (AUC/MIC > 100). For patients with Ccr ≤ 30 mL/min, the author recommends 300 mg q12h (49). A study in Japan showed that among patients with renal insufficiency, the clinical failure rate was significantly lower among linezolid-treated patients in the TDM group than in the non-TDM group. In addition, up to 90.5% of patients in the TDM group required dose adjustment. Patients with renal insufficiency can reach the goal of C_min_ ≥ 2.0 mg/L with a 300 mg q12h regimen (18). Kawasuji et al. (50) reported that the initial dose should be reduced to reduce the risk for linezolid-induced thrombocytopenia (LIT) in patients on HD.

In addition, the recommendation in the linezolid instructions that “dose does not need to be adjusted for patients with renal insufficiency” is mainly based on an early PK study. The study included only healthy volunteers (*n* = 6), individuals with moderately impaired renal function (*n* = 6), individuals with severely impaired renal function (*n* = 6), and patients on HD (*n* = 6). The subjects were given a single oral dose of linezolid 600 mg, and there were no significant differences in linezolid PK parameters (51). We have reason to believe that the recommendations in the instructions do not reflect the clinical situation. In a meta-analysis of the relationship between renal function and LIT, renal function was closely related to LIT, and poor renal function was related to a higher risk for LIT. Subgroup analyses showed that in patients on the conventional linezolid regimen (600 mg q12h), the risk for LIT was higher in patients with renal insufficiency than in those with normal renal function (unadjusted OR: 2.59, 95% CI: 1.64–4.10, *I*^2^ = 60.8% vs. adjusted OR: 2.69, 95% CI: 1.83–3.95, *I*^2^ = 0%). Therefore, the dosage recommended in the instructions is unsuitable (52).

A prospective, single-dose pharmacokinetic study included 15 critically ill patients with oliguria and acute renal insufficiency, eight of whom received intermittent HD lasting 3 to 4 h and five of whom received sustained low-efficiency dialysis (SLED) for 8 h. The patients received linezolid 600 mg intravenously within 60 min before dialysis. The average clearance rates of HD and SLED were 32.3 and 33.9%, respectively. After HD and SLED, the serum linezolid concentration in three patients was <4 mg/L (lower than the target MIC). To maintain efficacy, it is recommended that linezolid be used at the end of dialysis (53). A prospective, multi-dose pharmacokinetic study included five critically ill patients with renal failure on intermittent hemodialysis (IHD) who received standard doses of linezolid intravenously. IHD not only reduced the serum linezolid concentration but also delayed a steady-state linezolid concentration. It is recommended that overweight patients and early dialysis patients on linezolid be given one third the full dose or a loading dose of 1,200 mg after the end of dialysis (54). In critically ill patients with continuous veno-venous hemodialysis (CVVHD) and continuous veno-venous hemodiafiltration (CVVHDF) on renal replacement therapy, the standard linezolid dosage (600 mg q12h) is given intravenously, the therapeutic concentration is insufficient, and the target cannot be achieved for pathogens with MIC ≥ 2 mg/L (55, 56). A prospective, single-center observational study in China included 40 patients with sepsis who received continuous renal replacement therapy (CRRT). The standard intravenous dose for patients on renal replacement therapy yielded a steady-state concentration effective against pathogens with an MIC of 2 mg/L. The dose group reached the PK/PD target, whereas the high-volume hemofiltration (HVHF) group did not. For MIC > 2 mg/L, neither group achieved the PK/PD target. It is recommended that patients with sepsis receive different doses of CRRT, which should be adjusted according to the TDM results (57). In patients with severe sepsis who receive CRRT, underexposure to linezolid is common; for MIC > 4 mg/L in particular, the conventional dose does not result in optimal exposure and so must be increased (58, 59).

We recommend that patients with renal insufficiency on RRT be monitored for insufficient linezolid exposure. The typical dosage (600 mg q12h) may not be efficacious. Therefore, we recommend monitoring the serum concentration of linezolid in such patients.

### Question 7: Does the linezolid dose need to be adjusted for patients with hepatic insufficiency?

#### Expert panel recommendations

We do not recommend adjusting the linezolid dose for patients with mild to moderate hepatic insufficiency (Child-Pugh grade A or B). (Score: 8.65)

### Question 8: How should the linezolid dose be adjusted for patients with hepatic insufficiency?

#### Expert panel recommendations

We recommend reducing the dose of linezolid in Child-Pugh grade C patients with severe hepatic insufficiency. (Score: 8.40)

#### Reason

The updated instructions for linezolid tablets (released June 22, 2020) and the updated drug instructions for linezolid glucose injection (September 1, 2020) indicate that there is no need to adjust the dose for patients with mild to moderate hepatic insufficiency. A single-center, retrospective, observational case-control 1:1 study by Luque et al. (41) included 52 patients receiving linezolid 600 mg q12h, of whom 26 were patients with liver cirrhosis (1, 13, and 12 cases of Child-Pugh grade A, B, and C, respectively). The median linezolid trough concentration (C_min, ss_) of patients with liver cirrhosis was 20.6 (17.4) mg/L, which was higher than the control group (2.7 [11.3] mg/L, *P* < 0.001). The median peak concentration (C_max, ss_) was 34.1 (22.7) mg/L, which was higher than the control group [16.5 (11.6) mg/L, *P* = 0.001]. The steady-state C_min_ values of linezolid are similar in patients with different degrees of liver cirrhosis [for Child-Pugh grades A, B, and C: 25.9, 16.2 (15.5), and 22.7 (25.5) mg/L, respectively, *P* = 0.547]. A prospective, open-label, uncontrolled study by Sasaki et al. examined the PK of linezolid in Japanese patients, including four with severe liver cirrhosis (Child-Pugh grade C). The clearance rate at a dosage of 600 mg q12h decreased significantly (~50% decrease) and showed higher C_min, ss_ values of 32.5, 36.4, 40.8, and 45.4 mg/L, respectively, which suggests changes in liver cirrhosis. Therefore, the PK of linezolid is different in patients with liver cirrhosis with different grade (49).

Hepatic insufficiency reduces linezolid clearance in critically ill patients (60). The effects of severe liver dysfunction (Child-Pugh grade C) on the PK of linezolid are unclear. A prospective study by Zhang et al. included 163 concentration samples from 45 patients with liver insufficiency. Their PPK model showed that PTA and CLcr were positively correlated with the linezolid clearance rate (CL), which confirms that liver insufficiency is an important factor in linezolid PK and dose optimization. Monte Carlo simulation was used to optimize the linezolid dose for patients with liver insufficiency (61). Simulation based on the final model showed that when the MIC was 2 μg/mL, for patients with a PTA of 20–40% or CLcr <10 mL/min, the 300 mg q12h dosage was safe and effective based on probabilities of AUC_0−24_: MIC ratio ≥ 80 of 92.9% and 96.9% and probabilities of a C_min, ss_ of 2–8 μg/mL of 99.3% and 99.4%. When the MIC was 2 μg/mL, for patients with PTA ≤ 20% a 400 mg q24h dosage was sufficient to achieve the therapeutic target (the probability of AUC_0−24_:MIC ratio ≥ 80 was 97.9%). When the MIC was 4 μg/mL, for patients with PTA ≤ 20%, at a 300 mg q12h dosage the probability of AUC_0−24_:MIC ratio ≥ 80 was 91.9%, that of C_min, ss_ > 8 μg/mL was 0.2%, and that of a C_min, ss_ of 2–8 μg/mL was 99.8%. A prospective, open-label, uncontrolled study by Sasaki et al. included four patients with severe liver cirrhosis (Child-Pugh grade C). Based on a model simulation, for patients with insufficient renal function (CLcr ≤ 30 mL/min) or severe liver cirrhosis (Child-Pugh grade C), it is recommended that the dosage be reduced to 600 mg/day (49). Wicha et al. used the maximal liver function capacity (LiMAx test) tool for linezolid dose adjustment in patients with hepatic insufficiency. They suggested that TDM should be performed for LiMAx <100 μg/kg/h to reduce the risk of excessively high target plasma concentrations (62, 63).

### Question 9: Does the linezolid dose need to be adjusted for patients on extracorporeal membrane oxygenation (ECMO)?

#### Expert panel recommendations

We recommend optimization of the linezolid dosage for patients on ECMO. (Score: 8.75)

### Question 10: How should linezolid administration be optimized for patients on ECMO?

#### Expert panel recommendations

We recommend that for patients on ECMO the following dosing schedule be used:

- MIC ≤ 1 mg/L: 600 mg q12h.
- MIC = 2 mg/L: 600 mg q8h.
- MIC > 2 mg/L: might require more than 4-fold the conventional dosage to achieve the PK/PD target. The safety of this regime is unknown, so we recommend a switch to other sensitive antibiotics.

Whichever dosage is used, it should be adjusted based on the results of TDM. (Score: 8.40).

#### Reason

To date, two PK studies of linezolid in patients on ECMO have been published. The case series reported by De Rosa et al. (64) in 2013 comprised three patients on 600 mg q12h linezolid, who had AUC_0−24*h*_ values of 212.58, 165.65, and 100.59 mg·h/L, respectively. For pathogens with an MIC of 1, the AUC/MIC target was reached; for pathogens with an MIC of 2 mg/L, one patient failed to achieve the target; and for pathogens with an MIC of 4 mg/L, the AUC/MIC target was not reached. Therefore, individualized regimens should be used for patients on ECMO. The PK of a patient infected after right lung transplantation reported by Nikolos et al. (65) differed significantly from that reported by De Rosa et al. After reaching steady state, the peak and trough linezolid concentrations were 1.7 and 0.4 mg/L, and the AUC_0−24*h*_ was estimated at 21.6 mg/L. This is lower than reported by De Rosa et al. (64). Kühn et al. (66) evaluated 112 and 186 blood samples from patients with and without ECMO, respectively; 34.8% of patients on ECMO were underexposed to linezolid. In addition, the multivariate linear GEE method was used to evaluate the influence of clinical factors on blood antibiotic concentrations. The results showed that linezolid overexposure may have been related to ECMO duration (*P* < 0.05). Therefore, patients on ECMO receiving linezolid should be monitored for underexposure during early use and for overexposure during long-term use.

The four patients differed significantly, so TDM is needed for patients on ECMO. We believe that for bacteria with MIC ≤ 1 mg/L, the optimal PK/PD index can be achieved using the doses on the drug label. For bacteria with an MIC of 2 mg/L, 600 mg q8h linezolid is appropriate. In the case of MIC > 2 mg/L, achieving the PK/PD target is difficult, so we recommend switching to another sensitive antibiotic. The MIC_90_ of linezolid for *Staphylococcus aureus* in China (67) is 2 mg/L, so the empirical linezolid regimen is 600 mg q8h.

Few studies have evaluated linezolid in ECMO patients, and those that have are small samples or case reports. Therefore, it is necessary to adjust the linezolid dose in patients on ECMO and perform TDM.

### Question 11: Do pediatric patients require linezolid dose optimization?

#### Expert panel recommendations

We recommend optimizing the linezolid dose for pediatric patients. (Score:9.60)

### Question 12: How should the dose of linezolid be adjusted for pediatric patients?

#### Expert panel recommendations

We recommend linezolid dose adjustment for pediatric patients based on TDM. (Score: 9.25)

#### Reason

Li et al. (68) conducted a prospective PK study on 112 pediatric patients ages 0–12 years and established a PPK model. If bacterial MIC ≥ 2 mg/L, the conventional dosage of 10 mg/kg q8h will lead to a high risk of underdosing in children, and the dosage should be increased to 15 or 20 mg/kg q8h. A single-center, prospective, open-label PPK study on critically ill children in China by Yang et al. showed that for MIC ≤ 1 mg/L, a dosage of 10 mg/kg q8h yields PTA > 96%. For MIC > 1 mg/L, the PTA was <70%. For an MIC of 2 mg/L and a dosage of 15 mg/kg q6h, the PTA increased from 63.6 to 94.6% (10). A retrospective study by Wang et al. (69) found that the rate of linezolid treatment for thrombocytopenia in pediatric ICU patients was 25.0% and was related to linezolid overexposure. Linezolid has a better effect in children with tuberculosis, but some children are overexposed to linezolid and the treatment is discontinued because of adverse events. This suggests the importance of linezolid dose optimization for children with tuberculosis (70–72). Cojutti et al. (9) reported that children need a higher linezolid dose for pathogens with MIC > 1 mg/L and that TDM should be encouraged to optimize linezolid exposure. Linezolid TDM is useful for pediatric patients with sparse clinical data or PK changes. Adaptive feedback control and model-informed precision dosing use Bayesian algorithms and PK models to predict linezolid exposure. Linezolid TDM should be included in the dosage optimization workflow (45).

In summary, we support dose optimization in pediatric patients based on TDM.

### Question 13: Do obese patients need linezolid dose optimization?

#### Expert panel recommendations

Standard linezolid doses are not adequate for obese patients, and so dose adjustment is recommended. (Score: 9.30)

### Question 14: How should a dose optimization strategy be implemented for obese patients?

#### Expert panel recommendations

Adjustments to the linezolid dosage should be based on CrCL (CKD-EPI) estimates, and escalation to 600 mg q8h is not recommended because of an unacceptably high risk for thrombocytopenia (Table 1). (Score: 8.95)

**Table 1:** Algorithm for determining the optimal linezolid dosage for overweight and obese patients.

**CrCL_(CKD−EPI)_**	**Coagulase-Negative**	**S. aureus**	**Enterococcus spp**.
**(mL/min/1.73m^2^)**	**Staphylococci (CoNS)**	**(MRSA or MSSA)**	**(VSE or VRE)**
0–29	450mg q12h	600mg q12h	600mg q12h
30–59	450mg q12h	600mg q12h	600mg q12h
60–129	450mg q12h	600mg q12h	450mg q8h
130–200	600mg q12h	450mg q8h	450mg q8h

#### Reason

In a controlled clinical study, concentrations of linezolid in plasma and subcutaneous tissue decreased with increasing body weight, and the recommended dosage did not yield concentrations sufficient to kill bacteria with MIC ≥ 2 mg/L in obese patients (43). A phase I comparative clinical trial evaluated the PK and PD of a single intravenous fixed dose of linezolid compared to a weight-adjusted dose. A weight-adjusted dose of linezolid 10 mg/kg might be more appropriate than a fixed dose for obese patients (73). Body weight has a marked effect on linezolid clearance, and the PTA decreases with increasing weight. Moreover, standard linezolid dosing in obese patients with pneumonia caused by MRSA (MICs 1–4 mg/L) leads to an unacceptably low (near zero to 60%) PTA for patients <65 years old (74). Furthermore, a prospective study showed that standard linezolid doses might not be adequate for critically ill obese patients with severe skin and soft tissue infection (44). A retrospective study assessed the PPK and PD of linezolid in overweight and obese hospitalized patients (42). Totals of 352 trough (minimum) and 293 peak (maximum) linezolid concentrations from 112 patients were analyzed. Patients were on linezolid because of sepsis, nosocomial pneumonia, bone and joint infection, skin and soft tissue infection, and central nervous system (CNS) infection. Only the estimated creatinine clearance (using the Chronic Kidney Diseases Epidemiology formula, CrCL [CKD-EPI]) covariate improved the model fit. Dosage reduction to 450 mg q12h might be optimal for patients with coagulase-negative staphylococcal infection and CrCL (CKD-EPI) <30 mL/min/1.73 m^2^. Dosage escalation to 450 mg q8h may be optimal for patients with CrCL (CKD-EPI) ≥ 60 mL/min/1.73 m^2^. However, dosage escalation to 600 mg q8h is not recommended because of an unacceptably high risk for thrombocytopenia.

### Question 15: How should the linezolid dose be individualized for patients with tuberculosis?

#### Expert panel recommendations

We recommend 1-month treatment with linezolid 600 mg twice daily. For long-term treatment (if tolerated until the end of treatment), a maximum dosage of 600 mg once daily is recommended. Irrespective of the dosage, we recommend TDM of the AUC and determination of the MIC of *Mycobacterium tuberculosis* strains. The recommended target is AUC/MIC > 100. (Score: 9.05)

#### Reason

Although linezolid was recommended in the revised version of *World Health Organization Recommendations on the Treatment of Drug-Resistant Tuberculosis, 2020 Update*, the optimal dosage for tuberculosis needs to be determined (75). In the Nix-TB open-label clinical study, most participants required a reduction in dose or interruption of linezolid. Thirty-seven (34%) participants completed 26 weeks of linezolid without interruption, although they may have had a dose reduction, and 16 (15%) completed 26 weeks at a 1,200 mg total daily dose of linezolid with no interruptions or dose reductions (76). Although one study recommended a linezolid dosage for tuberculosis of 1,200 mg/day (75), others suggest that <600 mg/day is effective and has a treatment success rate similar to that of >600 mg/day (77). Indeed, 300 mg/day may have greater clinical efficacy with fewer adverse reactions. The optimal dosage is unclear (78–80). According to clinical studies, linezolid is appropriate for *M*. *tuberculosis* infection and can maintain a PK/PD target of AUC/MIC > 100 (81–86). Provision by the tuberculosis laboratory of the MIC of *M*. *tuberculosis* allows calculation of the AUC/MIC, potentially benefitting treatment. If the tuberculosis laboratory cannot provide the MIC of *M*. *tuberculosis*, it should estimate the AUC/MIC based on the local drug resistance profile of *M*. *tuberculosis* (87–91).

The problems with using linezolid for *M*. *tuberculosis* infection deserve further study. We suggest that the dosage be limited to 600 mg/day. Because of the lack of randomized controlled trials, we are unable to provide specific recommendations. However, we can reach a consensus on the TDM of linezolid for tuberculosis patients, and the target can also be determined at AUC/MIC > 100.

### Question 16: How should the linezolid dose be adjusted in patients with central nervous system (CNS) infection?

#### Expert panel recommendations

For CNS infection, we recommend the linezolid dosage as reported in the package insert (i.e., a linezolid dosage of 600 mg q12h in adults and children >12 years with normal renal and hepatic function). The recommended linezolid dosage is 10 mg/kg q8h in children up to 11 years of age and 10 mg/kg q12h in preterm (gestational age: 34 weeks) and 7-day-old infants. (Score: 8.65)

#### Reason

Beer et al. studied the PK profile of linezolid in CSF in five adult patients with staphylococcal ventriculitis. The patients received linezolid 600 mg q12h. The mean area under the concentration-time curve in CFS was 63 ± 18.9 mg·h/L with a CSF:plasma ratio of 0.8 ± 0.3. The proportion of time above the MIC in CSF was 99.8% and 57.2% for pathogens with MICs of 2 and 4 mg/L, respectively (92).

Yogev et al. (93) performed two studies in hydrocephalic children and adolescents to assess linezolid penetration of CSF. Patients 12 months to 24 years of age in study 1 and neonates <12 years of age in study 2 were administered intravenous linezolid 10 mg/kg q12h for 3 days (study 1) or q8h for 2 days (study 2). PK indices were determined for plasma and ventricular fluid (VF) after the first and last doses. In study 1, after the last dose, the mean C_max_ and C_min_ values for VF were 7.54 μg/mL (range: 2.26–12.6 μg/mL) and 1.26 μg/mL (range: 0.19–2.58 μg/mL), respectively. The VF:plasma ratio based on the last dose AUC_0−12_ was 0.98 μg·h/mL (range: 0.64–1.22 μg·h/mL). In study 2, after the last dose, the mean VF C_max_ and C_min_ values were 5.84 μg/mL (range: 1.82–9.34 μg/mL) and 1.94 μg/mL (range: 0.34–4.62 μg/mL), respectively. The VF:plasma ratio based on last dose AUC_0−8_ was 0.95 μg·h/mL (range: 0.62–1.31 μg·h/mL). Therefore, systemic clearance of linezolid decreases with age up to 12 years. However, some case reports suggest that the standard dose of linezolid has a better drug distribution in the brain and is more effective against common CNS pathogens (94–100).

In short, use of linezolid for intracranial infection is beyond the approved indications in the drug insert, and there are no RCTs on linezolid for CNS infection. Further PK/PD studies of linezolid in the CNS are needed.

### Question 17: How should adverse reactions to linezolid be monitored?

#### Expert panel recommendations

(1) We recommend that during linezolid treatment, platelets be monitored to detect hematological toxicity. (Score: 9.35) (2) We recommend pharmaceutical care for patients who are elderly, have renal insufficiency, have baseline thrombocytopenia, and have low body weight in whom long-term continuous (>1 month) use of linezolid achieves a trough concentration > 8 mg/L. (Score: 9.40)

### Question 18: How should linezolid-associated hyperlactacidemia be prevented and treated?

#### Expert panel recommendations

(1) We recommend monitoring blood linezolid and lactic acid levels while using linezolid. (2) Linezolid should not be used in combination with drugs that affect mitochondrial function. (3) Patients with liver or renal dysfunction, or those treated with linezolid for >1 month, should be monitored for hyperlactacidemia. (4) If hyperlacticacidosis occurs, we recommend stopping linezolid and correcting the acidosis as soon as possible. (Score: 9.35)

#### Reason

A retrospective study by Guo et al. included 5,336 patients on linezolid, of whom 266 (4.99%) had drug-induced thrombocytopenia. The incidence of thrombocytopenia increased significantly with age. Age was a significant risk factor for linezolid-related thrombocytopenia (OR: 4.887, 95% CI: 3.958–6.035, *P* = 0.000). Thrombocytopenia occurred within 7 days of medication in 45.26% of patients, and the decrease was greater than in other groups (101). Takahashi et al. performed regression tree analyses of 74 patients; the cutoff value for intervention was a reduction in platelet count to <2.3% that at baseline or a trough linezolid concentration ≥ 13.5 mg/L 96 h after initial administration. These cutoff values will appear before the onset of thrombocytopenia, and proactive monitoring can avoid LIT (102). A retrospective study by Morata et al. included 104 patients treated with linezolid; 34.6% had C_min_ > 8 mg/L, and more patients had GFR <40 mL/L by Modification of Diet in Renal Disease (MDRD). The only factor independently related to C_min_ > 8 mg/L was renal function. The C_min_ of patients with eGFR <40 mL/min was significantly higher than that of patients with eGFR > 80 mL/min (OR: 4.273), whereas the C_min_ of patients with an eGFR of 40–80 mL/min tended to be higher (OR: 2.109) (48). The hematological toxicity of linezolid in elderly patients with renal insufficiency may be related to drug overexposure (14, 16, 39, 40, 48–50).

An observational study by Niwa et al. (12) showed that a body weight <55 kg (OR: 33.2, 95% CI: 2.16–510.1, *P* = 0.012) and a baseline platelet count <200 × 10^3^/mm^3^ (OR: 24.9, 95% CI: 1.53–404.7, *P* = 0.024) are risk factors for LIT. In subsequent intervention studies, the daily dosage of linezolid was set to 1,200 mg/kg for patients with one or no risk factors. The incidence of thrombocytopenia in the intervention study group was significantly prolonged without a reduction in clinical efficacy (103).

Lactic acidosis is a rare but serious side effect of linezolid. From August 2011 to August 2016, 63 cases (27 deaths) and 243 cases (37 deaths) of linezolid-related lactic acidosis were reported by the U.S. FDA and the European Drug Reaction Report system, respectively (104–106). Based on these data, approximately 50 cases of linezolid-related lactic acidosis occur annually, with a mortality rate of 15% (37/243) to 43% (27/63) (104). Three retrospective studies reported an incidence of linezolid-associated hyperlactacidemia of 2–3% (107–109). A prospective study found that the incidence of lactic acidosis was 33% (5/15) after linezolid treatment for >1 month (110). Therefore, the incidence of linezolid-associated lactic acidosis may increase with treatment for >1 month. Case reports suggest that lactic acidosis occurs 1–16 weeks after linezolid administration (median: 5.5 weeks) (111).

The mechanism of linezolid-associated hyperlactacidemia is unclear, but linezolid has an antibacterial effect by binding to the bacterial ribosome subunit. Bacterial ribosomes have a structure similar to that of mitochondria. Linezolid interacts with mitochondrial ribosomes and affects mitochondrial translation activity, leading to hyperlactacidemia (104, 105, 107, 109, 111). The linezolid concentration required for binding to human mitochondrial ribosomes exceeds that required for therapeutic efficacy (111). Genetic polymorphisms of human mitochondrial 16S rRNA (such as A2706G and G3010A) may increase the risk of toxicity (104). During linezolid therapy, the risk for hyperlactacidemia is elevated by the use of other drugs that interfere with mitochondrial function (e.g., propofol, antiretrovirals, omeprazole, amiodarone, and amlodipine) (104).

Linezolid is metabolized mainly in the liver. Clearance of linezolid is reduced in patients with severe cirrhosis (Child-Pugh grade C) (48, 112). More than 60% of lactic acid is converted into pyruvate in the liver for gluconeogenesis (104). The serum linezolid concentration in patients with liver dysfunction is 4- to 6-fold that of patients with normal liver function (113). The incidence of lactic acidosis is higher in patients with liver function because of the excessive blood concentration of linezolid and lactic acid accumulation (104, 114).

Approximately 30% of linezolid is eliminated by the kidneys, but PK studies have shown that linezolid accumulation is not common in patients with renal dysfunction. Therefore, renal failure is not a major risk factor for linezolid-induced acidosis (51, 109). The kidneys metabolize about 30% of total lactic acid. Although severe renal dysfunction is unrelated to the incidence of linezolid acidosis, it does increase its severity and mortality (109). Discontinuation of linezolid is the most effective treatment for linezolid-associated hyperlactacidemia, and most patients recover in 1–15 days (104, 109, 115). There are no large, randomized cohort studies of RRT for linezolid acidosis. Clinical experience confirms that RRT is beneficial for patients with lactic acid excretion and can be used to correct acidosis (104, 109). When the enzyme formed by vitamin B1 and pyrophosphate is deficient, oxidative metabolism of sugars is blocked, and pyruvate and lactic acid accumulate (114, 116). Vitamin B1 should be supplied to patients with vitamin B1 deficiency on linezolid therapy (117).

## Summary

Overview of expert consensus and summary of recommendations in Table 2. There is doubt about the clinical use of linezolid. We hope that this expert consensus will promote individualized, safe, and rational use of linezolid in clinical practice. Because knowledge always lags evidence, this expert consensus has limitations, which we aim to overcome in subsequent editions.

**Table 2:** Overview of expert consensus and summary of recommendations.

**Numbers**	**Questions**	**Recommendations**
1	Does linezolid require TDM? What is the target range for linezolid TDM?	(1) We recommend TDM for linezolid. (Score: 8.90) (2) We recommend maintaining a linezolid trough concentration of 2–8 mg/L. (Score: 8.60)
2	What detection method is recommended for TDM of linezolid?	We recommended quantification of linezolid in human plasma or serum by HPLC-UV or LC-MS/MS methods. (Score: 8.80)
3	How should laboratories conducting quantitative determination of linezolid perform quality control?	Quality control procedures should be implemented using drug *in vivo* analysis techniques, quality control standards, and clinical intervention programs. (Score: 9.15)
4	How should linezolid TDM results be reported?	We recommend that the TDM report provide test results, explanations thereof, and treatment suggestions. The report should be individualized for the patient in question. (Score: 9.15)
5	For which patients should linezolid TDM be considered?	We recommend TDM for critically ill patients, children, patients with renal insufficiency/augment or liver cirrhosis, elderly, obese and patients taking co-medications known to interact with linezolid. (Score: 9.50)
6	How should the linezolid dosage be adjusted for patients with renal insufficiency?	(1) For patients with renal insufficiency not on hemodialysis (HD), we suggest that linezolid can be reduced to a regimen of 300 mg q12h. (Score: 7.90) (2) Insufficient linezolid doses in patients with renal insufficiency on dialysis must be considered, and the dose should be increased based on TDM if necessary. (Score: 8.70) (3) Irrespective of whether patients with renal insufficiency are on dialysis, we recommend adjusting the dose of linezolid based on TDM. (Score: 8.15)
7	Does the linezolid dose need to be adjusted for patients with hepatic insufficiency?	We do not recommend adjusting the linezolid dose for patients with mild to moderate hepatic insufficiency (Child-Pugh grade A or B). (Score: 8.65)
8	How should the linezolid dose be adjusted for patients with hepatic insufficiency?	We recommend reducing the dose of linezolid in Child-Pugh grade C patients with severe hepatic insufficiency. (Score: 8.40)
9	Does the linezolid dose need to be adjusted for patients on ECMO?	We recommend optimization of the linezolid dosage for patients on ECMO. (Score: 8.75)
10	How should linezolid administration be optimized for patients on ECMO?	(1) We recommend that for patients on ECMO the following dosing schedule be used: (2) MIC ≤ 1 mg/L: 600 mg q12h (3) MIC = 2 mg/L: 600 mg q8h (4) MIC > 2 mg/L: might require more than 4-fold the conventional dosage to achieve the PK/PD target. The safety of this regime is unknown, so we recommend a switch to other sensitive antibiotics. (5) Whichever dosage is used, it should be adjusted based on the results of TDM. (Score: 8.40)
11	Do pediatric patients require linezolid dose optimization?	We recommend optimizing the linezolid dose for pediatric patients. (Score:9.60)
12	How should the dose of linezolid be adjusted for pediatric patients?	We recommend linezolid dose adjustment for pediatric patients based on TDM. (Score: 9.25)
13	Do obese patients need linezolid dose optimization?	Standard linezolid doses are not adequate for obese patients, and so dose adjustment is recommended. (Score: 9.30)
14	How should a dose optimization strategy be implemented for obese patients?	Adjustments to the linezolid dosage should be based on CrCL (CKD-EPI) estimates, and escalation to 600 mg q8h is not recommended because of an unacceptably high risk for thrombocytopenia (Table 1). (Score: 8.95)
15	How should the linezolid dose be individualized for patients with tuberculosis?	We recommend 1-month treatment with linezolid 600 mg twice daily. For long-term treatment (if tolerated until the end of treatment), a maximum dosage of 600 mg once daily is recommended. Irrespective of the dosage, we recommend TDM of the AUC and determination of the MIC of Mycobacterium tuberculosis strains. The recommended target is AUC/MIC > 100. (Score: 9.05)
16	How should the linezolid dose be adjusted in patients with CNS infection?	For CNS infection, we recommend the linezolid dosage in the instructions (i.e., a linezolid dosage of 600 mg q12h in adults and children > 12 years with normal renal and hepatic function). The recommended linezolid dosage is 10 mg/kg q8h in children up to 11 years of age and 10 mg/kg q12h in preterm (gestational age: 34 weeks) and 7-day-old infants. (Score: 8.65)
17	How should adverse reactions to linezolid be monitored?	(1) We recommend that during linezolid treatment, platelets be monitored to detect hematological toxicity. (Score: 9.35) (2) We recommend pharmaceutical care for patients who are elderly, have renal insufficiency, have baseline thrombocytopenia, and have low body weight in whom long-term continuous (>1 month) use of linezolid achieves a trough concentration > 8 mg/L. (Score: 9.40)
18	How should linezolid-associated hyperlactacidemia be prevented and treated?	We recommend monitoring blood linezolid and lactic acid levels while using linezolid. Linezolid should not be used in combination with drugs that affect mitochondrial function. Patients with liver or renal dysfunction, or those treated with linezolid for >1 month, should be monitored for hyperlactacidemia. If hyperlacticacidosis occurs, we recommend stopping linezolid and correcting the acidosis as soon as possible. (Score: 9.35)

## Author contributions

BL, HD, and SJ initiated and organized the writing of expert consensus. BL organization was coordinated and revised the manuscript. HD and SJ reviewed and revised the manuscript. All authors contributed to different sections and manuscript was submitted after the approval.

## Funding

This work was supported by the Huzhou Medical Key Discipline Construction Project (Clinical Pharmacy) and the Key Laboratory of Intelligent Pharmacy and Individualized Therapy of Huzhou.

## Conflict of interest

The authors declare that the research was conducted in the absence of any commercial or financial relationships that could be construed as a potential conflict of interest.

## Correction note

This article has been corrected with minor changes. These changes do not impact the scientific content of the article.

## Publisher's note

All claims expressed in this article are solely those of the authors and do not necessarily represent those of their affiliated organizations, or those of the publisher, the editors and the reviewers. Any product that may be evaluated in this article, or claim that may be made by its manufacturer, is not guaranteed or endorsed by the publisher.

## Author disclaimer

This expert consensus recommendation is based on a review of a series of clinical studies on linezolid. However, the recommendations may have certain limitations, and clinicians should carry out clinical practice in combination with local resources and laws and regulations on the extended application of drugs. The recommendations made in this expert consensus cannot completely replace the judgment of physicians, and this recommendation needs to be adopted in combination with specific clinical practice.
